# Temozolomide and ruxolitinib combination modulates miRNA-associated regulatory networks in glioblastoma stem cells

**DOI:** 10.1007/s00210-026-05506-3

**Published:** 2026-06-08

**Authors:** Neslihan Pinar Ozates, Aycan Asik, Bakiye Goker Bagca, Cigir Biray Avci

**Affiliations:** 1 Department of Medical Biology, Faculty of Medicine, Harran University, Sanliurfa, Turkey; 2 Department of Medical Biology, Faculty of Medicine, Mugla Sitki Kocman University, Mugla, Turkey; 3 Department of Medical Biology, Faculty of Medicine, Ege University, Izmir, Turkey

**Keywords:** MiRNA, Ruxolitinib, Temozolomide, Combination

## Abstract

Glioblastoma is an aggressive primary brain tumor characterized by high recurrence rates and resistance to standard therapies, including temozolomide (TMZ). Emerging evidence suggests that microRNAs (miRNAs) play a critical role in regulating treatment response and tumor progression. In this study, we investigated the effects of TMZ and the JAK inhibitor ruxolitinib, alone and in combination, on miRNA expression profiles in glioblastoma cancer stem cells. miRNA expression levels were analyzed using quantitative real-time PCR, and differential expression was evaluated using the 2^−ΔΔCt^ method. TMZ treatment increased the expression of oncogenic miRNAs, including hsa-miR-19a-3p, hsa-miR-221-3p, and hsa-miR-10a-5p, whereas ruxolitinib treatment reduced their expression levels. Combination treatment attenuated the TMZ-induced upregulation of these miRNAs. Bioinformatics analyses were performed to identify target genes and associated signaling pathways. Predicted targets were obtained using TargetScan and further supported by experimentally validated interactions from miRTarBase. KEGG pathway enrichment analysis revealed significant associations with cancer-related pathways, including PI3K-Akt, MAPK, Wnt, and Hippo signaling pathways. Network analysis highlighted key genes such as BCL2L11, PTEN, and SOCS family members. Overall, our findings suggest that TMZ may induce oncogenic miRNA expression as part of an adaptive response, while ruxolitinib may counteract this effect. Targeting miRNA-mediated regulatory networks in combination with pathway inhibition may represent a promising strategy to overcome therapeutic resistance in glioblastoma.

## Introduction

Glioblastoma (GBM) is the most aggressive primary brain tumor in adults and is associated with a dismal prognosis. Despite the current standard of care, which includes surgical resection followed by radiotherapy and temozolomide (TMZ) chemotherapy, overall survival remains limited. The poor clinical outcome is largely attributed to high rates of tumor recurrence and the development of therapeutic resistance (Shetty et al. 2025; Singh et al. 2025).

Glioblastoma cancer stem cells (BCSCs) are increasingly recognized as key contributors to tumor recurrence and therapeutic resistance. These cells possess self-renewal capacity and exhibit enhanced resistance to conventional treatments, including increased DNA repair capabilities. By promoting tumor heterogeneity, BCSCs play a central role in disease progression and represent an important target for understanding treatment failure in glioblastoma (Alves et al. 2021).

Temozolomide (TMZ) is the main chemotherapeutic agent used in the treatment of glioblastoma; however, its clinical benefit is often limited by both intrinsic and acquired resistance mechanisms. In addition to this, growing evidence suggests that TMZ exposure can trigger adaptive cellular responses that enhance tumor cell survival and reduce long-term treatment efficacy (H. Li et al. 2025).

Dysregulation of signaling pathways such as JAK/STAT has been closely associated with glioblastoma progression, stem-like characteristics, and resistance to therapy. Ruxolitinib, a selective JAK1/2 inhibitor, has gained attention for its ability to modulate these pathways and interfere with tumor cell survival mechanisms. By targeting JAK/STAT signaling, ruxolitinib may contribute to improved therapeutic responses in glioblastoma (Ou et al. 2021; Sarapultsev et al. 2023).

MicroRNAs (miRNAs) are small non-coding RNAs that regulate gene expression at the post-transcriptional level and are involved in key cellular processes such as apoptosis, proliferation, and signaling pathway control. Dysregulated miRNA expression has been increasingly associated with tumor progression and resistance to therapy in glioblastoma (de Rooij et al. 2022; Evers et al. 2023).

Building on our previous study demonstrating that combined temozolomide (TMZ) and ruxolitinib treatment enhances apoptosis and modulates key signaling pathways in glioblastoma, we aimed to further investigate the underlying molecular mechanisms (Goker Bagca et al. 2022). In this study, we examined the effects of TMZ and ruxolitinib, both individually and in combination, on miRNA expression profiles in glioblastoma cancer stem cells. Additionally, target prediction and pathway enrichment analyses were conducted to identify potential regulatory pathways associated with treatment response.

## Materials and methods

### Cell culture

Commercially obtained brain cancer stem cells (BCSCs) (Celprogen, Cat. No. 36110–37) were used as a cancer stem cell model in this study. Cells were cultured in the medium recommended by the manufacturer and maintained in a humidified incubator at 37 °C with 5% CO_2_. Cells at approximately the eighth passage were used for all experiments (Goker Bagca et al. 2022).

### Reagents and treatment preparation

Ruxolitinib (Incyte Pharmaceuticals) and temozolomide (Sigma-Aldrich, USA) were dissolved in dimethyl sulfoxide (DMSO) to prepare stock solutions at concentrations of 10 mM and 50 mM, respectively. These stock solutions were further diluted with appropriate culture medium to obtain the desired working concentrations. Based on our previous study, the IC_50_ values of ruxolitinib and temozolomide were determined to be 94.07 μM and 512.1 μM, respectively, following 24 h of treatment. These concentrations were used to prepare the working solutions and were applied to the cells in subsequent experiments (Goker Bagca et al. 2022).

### miRNA expression analysis

To evaluate the effects of temozolomide (TMZ), ruxolitinib, and their combination on miRNA expression levels, total RNA, including small RNAs, was isolated from treated and control brain cancer stem cells using the RNeasy Mini Kit (Qiagen, Germany), following the manufacturer’s instructions. Complementary DNA (cDNA) was synthesized using the miScript II RT Kit (Qiagen, Germany), and miRNA expression analysis was performed by quantitative real-time PCR (qRT-PCR) using the miScript SYBR Green PCR Kit (Qiagen, Germany). Analyzed miRNAs were selected based on their potential roles in cancer-related processes, and their primer information is presented in Table 1. SNORD61 was used as the endogenous control for normalization according to the recommendations of the Qiagen miScript system.

**Table 1:** miRNA primer information used for expression analysis. List of miRNAs analyzed in this study along with their corresponding GeneGlobe IDs and catalog numbers. All primers were obtained from Qiagen (miScript Primer Assays) and used for quantitative real-time PCR (qRT-PCR) analysis. SNORD61 was used as an internal reference control for normalization.

	**Product name**	** GeneGlobe ID**	** Catalog number**
* 1*	hsa-let-7e-3p	YP00205301	339306
* 2*	hsa-miR-106a-3p	YP00204443	339306
* 3*	hsa-miR-10a-5p	YP00204778	339306
* 4*	hsa-miR-126-3p	YP00204227	339306
* 5*	hsa-miR-17-3p	YP00206008	339306
* 6*	hsa-miR-19a-3p	YP00205604	339306
* 7*	hsa-miR-21-3p	YP00204302	339306
* 8*	hsa-miR-221-3p	YP00204532	339306
* 9*	hsa-miR-708-5p	YP00204490	339306
*10*	HS_SNORD61_1531892	SBH0288097	249990

Relative expression levels were calculated using the 2^−ΔΔCt^ method after normalization to internal control small RNAs. Data analysis and heatmap generation were performed using the Qiagen GeneGlobe Data Analysis Center (https://geneglobe.qiagen.com).

### Statistics

All experiments were performed in triplicate, and data were expressed as mean ± standard deviation. Statistical analysis was conducted using Qiagen GeneGlobe Data Analysis Center (https://geneglobe.qiagen.com) software. The *p*-values are calculated based on a Student’s *t*-test of the replicate 2^(− delta Ct) values for each gene in the control group and treatment groups, and *p*-values < 0.05 are indicated in red.

### Bioinformatics analysis

Predicted target genes of selected miRNAs were identified using the TargetScan database. To further support the reliability of the predicted interactions, experimentally validated miRNA–target interactions were also examined using the miRTarBase database. Only Homo sapiens targets were included, and duplicate genes were removed prior to downstream analyses. Functional enrichment analysis was performed using the DAVID platform (v6.8) to identify significantly associated Kyoto Encyclopedia of Genes and Genomes (KEGG) pathways. A *p*-value < 0.05 was considered statistically significant. In addition, miRNA–target gene interaction networks were constructed and visualized using Cytoscape software (Q. Li et al. 2019).

## Results

### miRNA expression analysis

**Fig. 1:**
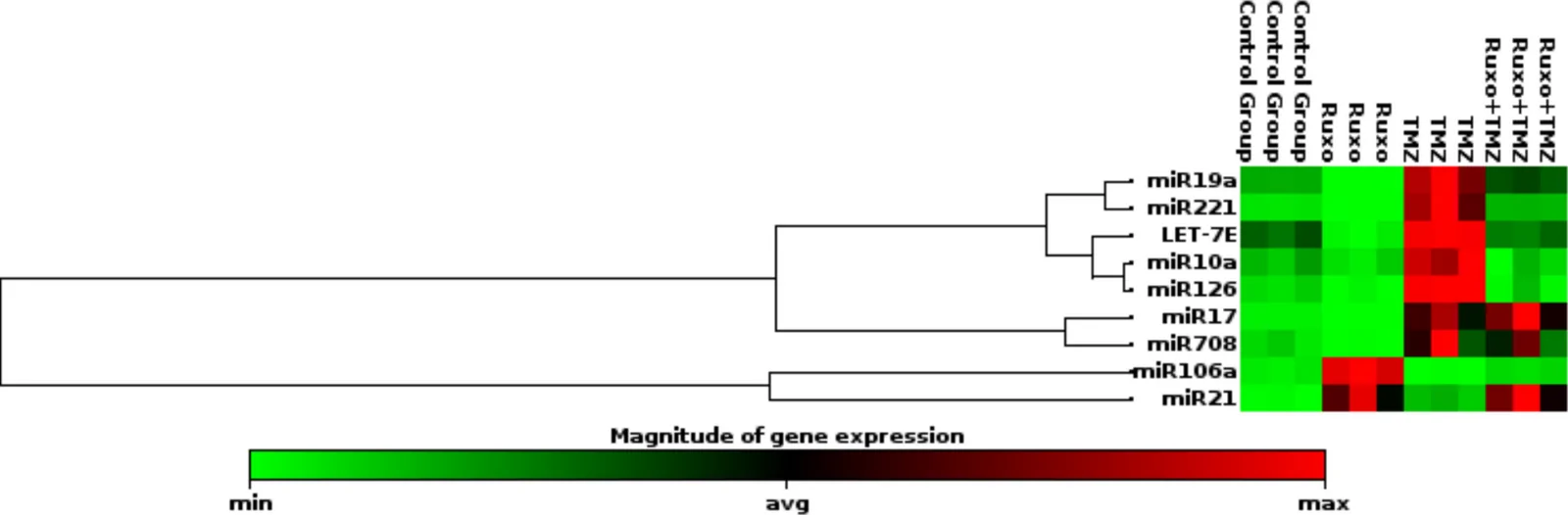
Heatmap of miRNA expression profiles in glioblastoma cancer stem cells following treatment. Heatmap illustrating the relative expression levels of selected miRNAs in control, temozolomide (TMZ), ruxolitinib (Ruxo), and combination (Ruxo + TMZ) treatment groups. Expression levels were normalized to internal reference controls and calculated using the 2^−ΔΔCt^ method. Hierarchical clustering was performed based on expression similarity, and both miRNAs and treatment groups were clustered accordingly. The color scale represents relative expression levels, where red indicates upregulation and green indicates downregulation. Data represent mean ± SD of three independent experiments

The expression profiles of selected miRNAs were evaluated in glioblastoma cancer stem cells following treatment with ruxolitinib, temozolomide (TMZ), and their combination. Relative expression levels were calculated using the 2^−ΔΔCt^ method and visualized using the Qiagen GeneGlobe Data Analysis Center. The heatmap analysis demonstrated distinct expression patterns across treatment groups, indicating that both single and combination treatments modulate miRNA expression profiles (Fig. 1).

Consistent with these findings, fold change analysis revealed that several miRNAs were significantly dysregulated in response to treatments (Table 2). Notably, differential expression patterns were observed between TMZ and combination treatment groups, suggesting a modulatory effect of ruxolitinib on miRNA expression. TMZ treatment increased the expression of oncogenic miRNAs such as hsa-miR-19a, hsa-miR-221, and hsa-miR-10a, while ruxolitinib reduced their expression. Combination treatment attenuated the TMZ-induced increase.

**Table 2:** Relative expression levels of selected miRNAs in response to treatments. Fold change and fold regulation values of miRNA expression levels in glioblastoma cancer stem cells treated with ruxolitinib, temozolomide (TMZ), and their combination (Ruxo + TMZ), compared to control. Expression levels were calculated using the 2^−ΔΔCt^ method. Fold regulation indicates upregulation (positive values) or downregulation (negative values). Statistical significance was determined using Student’s *t*-test based on replicate 2^−ΔCt^ values. *p*-values less than 0.05 are considered statistically significant.Bold entries indicate statistically significant values (p < 0.05).

	Ruxolitinib			Temozolomide			Ruxo + TMZ		
	Fold change	Fold regulation	***p***-value	Fold change	Fold regulation	***p***-value	Fold change	Fold regulation	***p***-value
***hsa-let-7e-3p ***	0.17	− 5.78	***0.0003***	2.92	2.92	***0.0000***	0.87	− 1.14	*0.1892*
***hsa-miR-106a ***	20.51	20.51	*0.0781*	0.18	0.18	***0.0052***	1.63	1.63	*0.2500*
***hsa-miR-10a***	0.56	− 1.80	***0.0000***	5.30	5.30	***0.0002***	0.53	− 1.90	***0.0327***
***hsa-miR-126***	0.11	− 8.86	***0.0074***	12.95	12.95	***0.0000***	0.47	− 2.13	*0.6689*
***hsa-miR-17***	0.01	− 170.70	***0.0000***	20.35	20.35	***0.0049***	24.01	24.01	***0.0059***
***hsa-miR-19a***	0.01	− 93.44	***0.0000***	5.28	5.28	***0.0009***	2.12	2.12	***0.0002***
***hsa-miR-21***	8.85	8.85	***0.0073***	2.42	2.42	***0.0022***	9.47	9.47	***0.0056***
***hsa-miR-221***	0.02	− 53.33	***0.0000***	15.54	15.54	***0.0011***	2.79	2.79	***0.0000***
***hsa-miR-708***	0.18	− 5.44	***0.0137***	7.00	7.00	***0.0452***	5.32	5.32	***0.0401***

### Prediction and validation of target genes

**Fig. 2:**
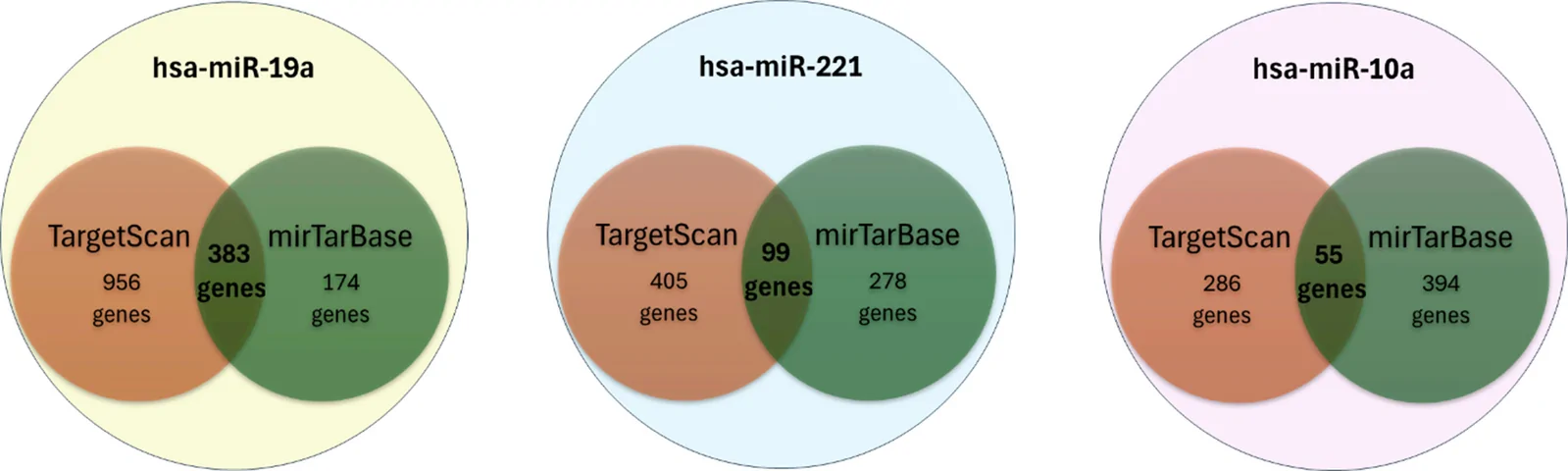
Comparison of predicted and experimentally validated target genes of selected miRNAs. Venn diagrams illustrating the overlap between predicted target genes identified using TargetScan and experimentally validated miRNA–target interactions obtained from the miRTarBase database for hsa-miR-19a, hsa-miR-221, and hsa-miR-10a. The number of unique and shared target genes for each miRNA is indicated. Overlapping genes represent high-confidence targets supported by both prediction algorithms and experimental validation, and were selected for subsequent pathway enrichment and network analyses

Predicted target genes of selected miRNAs were identified using the TargetScan database and further supported by experimentally validated interactions obtained from miRTarBase. Venn diagram demonstrated the overlap between predicted and validated target genes for each miRNA (Fig. 2). Among the analyzed miRNAs, hsa-miR-19a exhibited the highest number of overlapping target genes between the two datasets. The overlapping gene lists were considered high-confidence targets and are presented in Table 3. In addition, a subset of genes was identified as commonly targeted by all three miRNAs, suggesting potential shared regulatory mechanisms.

**Table 3:** Predicted and validated target genes of selected miRNAs. Target genes of hsa-miR-19a-3p, hsa-miR-221-3p, and hsa-miR-10a-5p identified using the TargetScan database and further supported by experimentally validated interactions from the miRTarBase database. The intersecting gene list represents high-confidence targets shared between prediction-based and experimentally validated datasets, which were used for subsequent pathway enrichment and network analyses. These genes were further used to construct miRNA–target interaction networks and perform functional enrichment analysis

miRNA	Target genes
hsa-miR-19a-3p	ABCA1, ABHD17C, ABHD5, ACBD5, ACSL4, ADIPOR2, ADSS, AFF1, AFTPH, AGO1, AGO3, AGPAT5, ALG2, AMMECR1L, ANKIB1, ANKRD12, ARAP2, ARC, ARFGEF1, ARHGAP1, ARHGAP12, ARHGEF26, ARIH2, ARL8A, ARMC8, ARPP19, ARRDC3, ASNA1, ATG14, ATG16L1, ATP6V1B2, ATXN1, ATXN7, AZIN1, BAMBI, BCL2L11, BCL3, BCL7A, BEND3, BLCAP, BMP3, BMPR2, BRWD1, BRWD3, BTBD7, BTF3L4, BTG1, C11orf57, C16orf70, C2orf42, C5orf24, C5orf30, CAB39, CALM1, CAMSAP1, CAMSAP2, CAPRIN2, CASZ1, CBX7, CC2D1A, CCNA2, CCND1, CCND2, CCNL1, CCNT2, CCSER2, CD164, CEP170, CEP350, CEP55, CFL2, CLIP1, CLOCK, CNOT6, CNOT7, COQ10B, CREBL2, CREBRF, CTGF, CUL5, DBN1, DCAF7, DCP2, DCUN1D3, DDX3X, DDX3Y, DDX6, DENND6A, DHX40, DICER1, DIP2A, DLG5, DMXL2, DUT, DYNC1LI2, E2F8, EFR3A, EIF4A2, ELL2, ELMOD2, ELOVL5, ENPP5, EOGT, EPN2, EPS15, ERBB4, ESR1, ETV3, EVI5L, FAM102A, FAM46C, FAM73A, FAM73B, FAM83D, FAS, FBXO10, FBXO28, FBXO48, FBXO8, FKBP15, FNDC3A, FOXP1, FRMD6, FRS2, FXR1, FZD6, G3BP2, GATAD2B, GFPT1, GIGYF1, GIT2, GMFB, GPR137B, GRB10, GRK6, GRSF1, GSKIP, HBP1, HDAC4, HECW2, HIC1, HIPK2, HIPK3, HNRNPF, HNRNPU, HNRNPUL1, HOMER1, HOXA5, HPRT1, IKZF1, IMPDH1, INO80, ITGA2, JARID2, JAZF1, KATNAL1, KCNJ2, KIAA0907, KIAA1468, KIF13A, KIF3A, KIT, KLF13, KLHL11, KLHL20, KLHL3, KLHL42, KPNA6, LCLAT1, LDLR, LIN9, LMLN, LONRF1, LZIC, MACF1, MAP2K3AQ, MAP3K1AQ, MAPK1AQ, MAPK14AQ, MB21D2, MBNL1, MBNL2, MBNL3, MCC, MECP2, MED12L, MEF2A, MFSD6, MID1IP1, MIER1, MKL2, MLEC, MLLT10, MMGT1, MOB1B, MPRIP, MRPL17, MSMO1, MTF2, MTMR12, MTMR6, MXD1, MYCN, NACC1, NACC2, NAPB, NF1, NFIA, NFIB, NPEPL1, NPTN, NRBF2, NRBP1, NUFIP2, NUP54, NUS1, OCRL, OTUD1, OTUD7B, PAPD4, PATL1, PCDH10, PDE4A, PFN1, PFN2, PGK1, PGM2L1, PHF13, PHLDA3, PIK3R3, PKNOX1, PLXNC1, PNRC1, PPARA, PPP1R15B, PPP2R5E, PPP6R1, PPTC7, PRICKLE2, PRKAA1, PRKACB, PRR14L, PRUNE2, PSAP, PTEN, PTPRG, PURG, QKI, RAB13, RAB14, RAB18, RAB1A, RAB21, RAB2B, RAB34, RAB5B, RAB8B, RACGAP1, RAF1, RAP1A, RAP1B, RAP2C, RAPGEF2, RAPGEF4, RASA1, RASSF2, RBBP8, RBM20, REEP3, RGL1, RHOB, RLIM, RNF11, RNF111, RNF167, RNF216, RNF44, RORA, RPS6KA5, RRAGD, S1PR2, SAMD8, SATB1, SBF2, SEC61A1, SEC63, SECISBP2L, SEMA4C, SERINC3, SESN3, SGK1, SH3KBP1, SHCBP1, SIVA1, SKIL, SLC12A7, SLC25A12, SLC27A1, SLC30A7, SLC37A1, SLC48A1, SLC6A8, SLC9A1, SLC9A6, SMAD5, SMARCA2, SMG1, SMOC1, SNX17, SOCS1, SOCS3, SOX4, SOX6, SPATA2, SPG20, SPTSSA, STK38, STOX2, STX12, SUZ12, TAF4, TBK1, TGFBR2, TGIF1, TGOLN2, THBS1, TMBIM6, TMEM106B, TMEM64, TNFAIP3, TNFRSF12A, TNFRSF1B, TNIP1, TNKS, TNPO2, TNRC6A, TNRC6B, TP53INP1, TRAK2, TRIM33, TRPC3, TXLNG, UBE2A, UBE2D3, UBL3, UBN2, USP13, USP37, USP8, VAMP3, VPS37A, VPS37B, VPS4B, WAC, WBP2, WBP4, WDFY3, WDR1, WDR20, WDR26, WDR45B, WNK1, WNK3, WNT10A, WNT7B, YY1, ZBTB18, ZBTB4, ZBTB47, ZBTB7B, ZDHHC18, ZDHHC7, ZFAND5, ZFYVE26, ZFYVE9, ZIC5, ZMAT3, ZMYND11, ZNF217, ZNF367, ZNF644, ZNF772, ZNF800
hsa-miR-221-3p	ARF4, ARHGAP42, ARID1A, ARNT, ATXN1, BBC3, BCL2L11, BMF, BRWD1, BRWD3, CAMKK1, CDKN1B, CDKN1C, CHSY1, CREB1, CREBZF, CTCF, DDIT4, DICER1, DKK2, ELAVL2, ERBB4, ESR1, ETS1, FBXO28, FMR1, FOS, GTF2E1, HECTD2, HMBOX1, HNRNPA0, KIF16B, KIT, KPNA2, LHFPL2, LYSMD1, MAP3K2AQ, MAPK10AQ, MIDN, MYBL1, MYLIP, NDFIP1, NUFIP2, OIP5, PAIP2, PAK1, PANK3, PCDHA1, PCDHA10, PCDHA11, PCDHA12, PCDHA13, PCDHA2, PCDHA3, PCDHA4, PCDHA5, PCDHA6, PCDHA7, PCDHA8, PCDHAC1, PCDHAC2, PDGFA, PDIK1L, PHF12, PIK3R1, PNRC2, POGZ, PPP1R15B, PPP6C, PTBP3, RAB1A, RECK, RNF4, RNF44, RNPS1, SNX4, SOCS1, SOCS3, SOCS5, SOX11, SPTSSA, SRSF2, STMN1, TFAP2A, THBS1, TIMP3, TIPARP, TMCC1, TMEM132B, TRPC3, TRPS1, UBE2J1, UBE2N, UBN2, WEE1, YOD1, ZBTB37, ZEB2, ZNF652
hsa-miR-10a-5p	ACTG1, AHSA2, BCL2L11, BCR, BDNF, BTRC, CADM1, CDC6, CHL1, CNOT6, CRK, CRLF3, CSRNP3, DVL3, E2F7, ELOVL2, EPHA4, EPHA8, FRS2, FZD2, GALNT1, GATAD2A, GPCPD1, H3F3B, H3F3C, HOXA1, HOXA3, HOXB3, IGSF1, LHFPL4, LIX1L, MAP3K7AQ, MED1, MTF2, NCOR2, NR2C2, NT5DC1, ONECUT2, PAPD5, PTEN, RAP2A, RORA, RPRD1A, SDC1, SERPINE1, SON, SRSF1, TFAP2A, TFAP2C, TIAM1, TRIM2, UBXN7, USF2, XRN1, ZFAND5
Overlapping genes	ATXN1, BCL2L11, BRWD1, BRWD3, CNOT6, DICER1, ERBB4, ESR1, FBXO28, FRS2, KIT, MTF2, NUFIP2, PPP1R15B, PTEN, RAB1A, RNF44, RORA, SOCS1, SOCS3, SPTSSA, THBS1, TRPC3, UBN2, ZFAND5

### Functional enrichment analysis

**Fig. 3:**
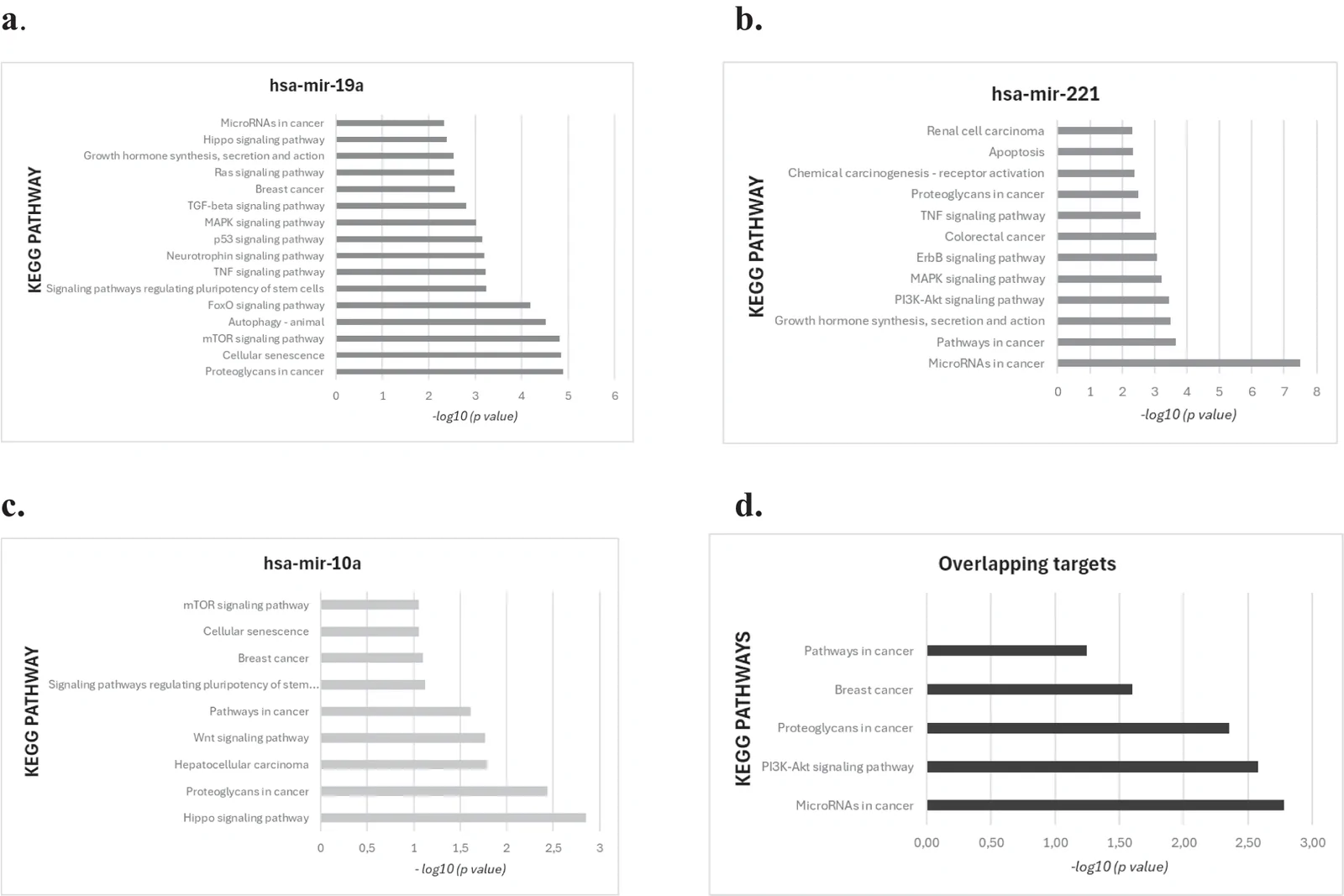
KEGG pathway enrichment analysis of miRNA target genes. **a.** Panel shows KEGG pathway enrichment of predicted target genes associated with hsa-miR-19a. **b.** Panel shows KEGG pathway enrichment of predicted target genes associated with hsa-miR-221. **c.** Panel shows KEGG pathway enrichment of predicted target genes associated with hsa-miR-10a. **d.** Panel shows KEGG pathway enrichment of overlapping target genes shared among the selected miRNAs. In all panels, the *x*-axis represents − log10 (*p*-value), indicating enrichment significance, while the *y*-axis lists the enriched KEGG pathways

Functional enrichment analysis of the high-confidence target genes was performed using the DAVID platform. KEGG pathway enrichment analysis revealed that the target genes of each miRNA were significantly associated with multiple cancer-related signaling pathways (Fig. 3).

For hsa-miR-19a, enriched pathways included MAPK, PI3K-Akt, FoxO, and mTOR signaling pathways, indicating involvement in cell proliferation and survival mechanisms. For hsa-miR-221, pathways such as apoptosis, TNF signaling, MAPK signaling, and PI3K-Akt signaling were significantly enriched, highlighting roles in apoptosis and inflammatory signaling processes. For hsa-miR-10a, key enriched pathways included Hippo signaling, Wnt signaling, and mTOR signaling pathways, suggesting a role in stemness and tumor progression. Furthermore, enrichment analysis of overlapping target genes identified common pathways such as PI3K-Akt signaling, proteoglycans in cancer, and pathways in cancer, indicating shared regulatory networks among the selected miRNAs.

### miRNA–target gene interaction network

**Fig. 4:**
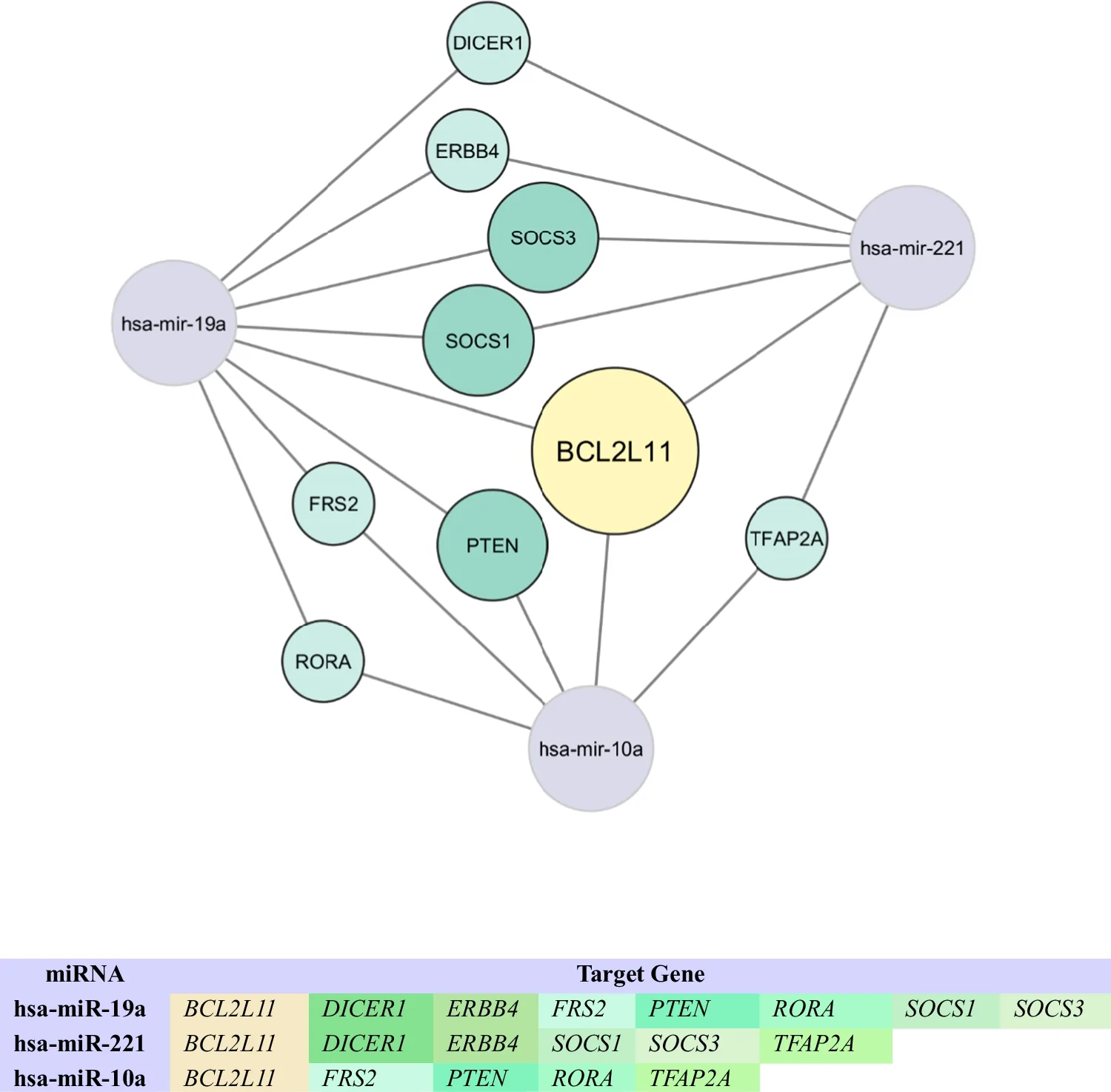
miRNA–target gene interaction network of selected miRNAs. Network representation of interactions between selected miRNAs (hsa-miR-19a, hsa-miR-221, and hsa-miR-10a) and their predicted target genes. Target genes were identified using TargetScan and further supported by experimentally validated interactions from the miRTarBase database. Nodes represent miRNAs and target genes, while edges indicate regulatory interactions. miRNAs are shown in purple, target genes are shown in light blue, and key hub genes are highlighted. Colors were selected to correspond with the associated interaction groups shown in the supplementary interaction summary table. Among these, BCL2L11 was identified as a central node associated with apoptosis, while PTEN was observed as a key tumor suppressor involved in PI3K-Akt signaling. Additionally, SOCS1 and SOCS3 were present in the network as regulators of the JAK/STAT signaling pathway. The network illustrates the coordinated regulation of cancer-related and apoptosis-associated pathways by multiple miRNAs

To further explore the interactions between miRNAs and their target genes, a network analysis was performed and visualized using Cytoscape. The constructed network revealed complex interactions between the selected miRNAs and multiple target genes (Fig. 4). Among these, BCL2L11 emerged as a central hub gene associated with apoptosis regulation, while PTEN was identified as a key tumor suppressor involved in PI3K-Akt signaling. Additionally, SOCS1 and SOCS3, known regulators of the JAK/STAT signaling pathway, were identified within the network, suggesting a potential mechanistic link with ruxolitinib treatment. Overall, the network analysis highlights the coordinated regulation of cancer-related and apoptosis-associated pathways by the selected miRNAs.

## Discussion

Glioblastoma (GBM) remains the most aggressive primary brain tumor, characterized by rapid recurrence and resistance to standard therapies, particularly temozolomide (TMZ). Despite multimodal treatment approaches, patient survival remains limited, largely due to intrinsic and acquired resistance mechanisms. TMZ resistance has been associated with multiple molecular processes, including enhanced DNA repair capacity, activation of pro-survival signaling pathways, and tumor heterogeneity (H. Li et al. 2025; Singh et al. 2025). A major contributor to this resistance is the presence of glioblastoma cancer stem cells (BCSCs), which possess self-renewal capacity and drive tumor heterogeneity and recurrence. These cells are highly resistant to conventional therapies and are capable of surviving TMZ treatment, thereby contributing to tumor relapse (Liu et al. 2023; Pasqualetti et al. 2023). Notably, recent studies suggest that TMZ treatment itself may promote stemness-related characteristics in glioblastoma cells by enabling a subset of cells to survive and acquire more aggressive phenotypes. This adaptive response further complicates treatment outcomes and highlights the importance of targeting stem cell-associated regulatory mechanisms in glioblastoma (Lan et al. 2023; Wang et al. 2025).

MicroRNAs (miRNAs) have emerged as important regulators of these adaptive responses because they can coordinately modulate multiple signaling pathways involved in proliferation, apoptosis, stemness, and therapy resistance. Consistent with this framework, our enrichment analyses identified significant associations between the selected miRNAs and cancer-related signaling pathways, including PI3K-Akt, MAPK, Wnt, Hippo, and mTOR signaling pathways (Sati & Parhar 2021). Furthermore, overlapping target analysis identified PTEN, BCL2L11, SOCS1, and SOCS3 as shared network components, suggesting that these miRNAs may participate in interconnected survival and inflammatory signaling networks rather than functioning independently.

In our previous study, we demonstrated that ruxolitinib potentiated TMZ-induced cytotoxicity and apoptosis in glioblastoma cells and stem cell–like populations, partly through modulation of Wnt-related signaling pathways. Building on these findings, the present study aimed to investigate whether miRNA-associated regulatory mechanisms might also contribute to the biological interaction between TMZ and ruxolitinib (Goker Bagca et al. 2022). Our results showed that TMZ treatment increased the expression of oncogenic miRNAs including hsa-miR-19a-3p, hsa-miR-221-3p, and hsa-miR-10a-5p, whereas ruxolitinib treatment reduced their expression. Importantly, combination treatment attenuated the TMZ-associated increase in these miRNAs. Rather than indicating direct mechanistic normalization, these findings suggest that ruxolitinib may partially counteract TMZ-associated adaptive miRNA remodeling in glioblastoma stem-like cells.

A major question raised by the present findings concerns the mechanistic relationship between JAK/STAT inhibition and miRNA regulation. Constitutive activation of STAT3 signaling is well established in GBM and has been associated with tumor proliferation, invasion, inflammatory signaling, and maintenance of stem cell-like phenotypes (Hu et al. 2026). IL-6-driven JAK/STAT3 signaling, in particular, has been shown to sustain glioma stem cell survival and self-renewal. Moreover, previous studies demonstrated that STAT3 can directly regulate specific oncogenic miRNAs in glioma biology. The best-characterized example is hsa-miR-21, whose transcription has been linked to STAT3-dependent signaling and glioma progression. These findings support the biological plausibility that JAK/STAT inhibition may influence miRNA expression programs in GBM (Rahaman et al. 2002).

However, for the miRNAs highlighted in the present study, hsa-miR-19a, hsa-miR-221, and hsa-miR-10a equivalent evidence demonstrating direct STAT3-dependent transcriptional regulation in glioblastoma remains limited. Therefore, the observed effects of ruxolitinib should be interpreted cautiously. Rather than concluding that these miRNAs are direct downstream targets of JAK/STAT signaling, it appears more plausible that ruxolitinib alters broader inflammatory, stemness-associated, and stress-response transcriptional programs, which secondarily reshape miRNA expression profiles. Potential mechanisms may include suppression of IL-6/NF-κB/STAT3 signaling, modulation of DNA damage-associated adaptive responses, and alterations in glioblastoma stem-like cell states. Accordingly, the present findings should be regarded as exploratory and hypothesis-generating rather than definitive mechanistic proof.

In our previous study, ruxolitinib enhanced TMZ-induced cytotoxicity and apoptosis in glioblastoma cells and glioblastoma stem cell populations, accompanied by alterations in signaling pathways associated with tumor survival and stemness. Given that ruxolitinib functions as a JAK1/2 inhibitor and suppresses STAT3-related signaling, the miRNA alterations observed in the present study may represent one potential regulatory layer underlying these previously reported biological effects. In particular, several target genes identified in our network analysis, including PTEN, BCL2L11, SOCS1, and SOCS3, are functionally linked to apoptosis, survival signaling, and JAK/STAT pathway regulation. Therefore, it is plausible that the cytotoxic and pro-apoptotic effects observed previously may be partially mediated through miRNA-associated regulatory mechanisms. However, direct mechanistic interactions between STAT3 inhibition and the regulation of the identified miRNAs remain to be experimentally validated.

Among the analyzed miRNAs, hsa-miR-221-3p showed the strongest translational relevance. miR-221/222 family members have consistently been associated with glioblastoma progression, invasion, anti-apoptotic signaling, and resistance to TMZ and radiotherapy. Several experimentally validated targets of hsa-miR-221, including PTEN, PUMA, p27/p57, EGFR, and SOCS3, have been implicated in treatment resistance and tumor survival. In particular, increased hsa-miR-221 expression has been associated with therapy-resistant GBM phenotypes and aggressive tumor behavior (Quintavalle et al. 2011; Schnabel et al. 2021). Our findings that TMZ increased hsa-miR-221 expression while ruxolitinib attenuated this increase are therefore consistent with previous reports suggesting that hsa-miR-221 contributes to adaptive resistance programs in glioblastoma. Importantly, SOCS3 was identified within our interaction network, which is noteworthy because miR-221/222-mediated suppression of SOCS3 has been reported to enhance JAK/STAT signaling activity (Yoshimura et al. 2007). Consequently, the reduction of hsa-miR-221 observed following ruxolitinib treatment may reflect disruption of a positive inflammatory feedback network rather than simple downstream inhibition of a STAT3-regulated miRNA (Areeb et al. 2020).

Similarly, hsa-miR-19a-3p, a member of the oncogenic miR-17–92 cluster, has been widely associated with glioma proliferation, invasion, and poor prognosis through regulation of PTEN, RUNX3, and β-catenin-related pathways. Previous studies demonstrated that hsa-miR-19a expression increases with glioma grade and contributes to pro-survival signaling networks (Shea et al. 2016). Consistent with these reports, TMZ-induced upregulation of hsa-miR-19a in our model may reflect an adaptive stress-response mechanism associated with therapy resistance. The attenuation of this increase by ruxolitinib may therefore indicate suppression of inflammatory or stemness-associated transcriptional programs linked to JAK/STAT signaling. The identification of PTEN and BCL2L11 in our network analysis further supports the functional relevance of hsa-miR-19a in apoptosis and survival regulation.

The findings related to hsa-miR-10a-5p are also noteworthy. Previous studies have linked hsa-miR-10a to acquired TMZ resistance, recurrent GBM phenotypes, epithelial-to-mesenchymal transition-like programs, and invasive behavior (Yan et al. 2015). Moreover, hsa-miR-10-related signaling has been associated with the regulation of pathways involved in tumor growth and stemness (Chen et al., 2023). Increased hsa-miR-10a expression has been reported in TMZ-resistant glioblastoma cells, and suppression of hsa-miR-10a has been shown to enhance TMZ sensitivity in experimental models (Ujifuku et al. 2010). In addition, hsa-miR-10a-associated networks have been connected to stemness-related signaling pathways, including Wnt and Hippo signaling, both of which emerged in our enrichment analyses. Therefore, the observed increase in hsa-miR-10a following TMZ treatment and its suppression under combination treatment are biologically consistent with previous resistance-associated observations.

To further investigate the functional roles of these miRNAs, their predicted and experimentally validated target genes were analyzed, and their interactions were visualized through network analysis. The constructed network revealed that PTEN and BCL2L11 serve as central nodes connecting multiple miRNAs, highlighting their potential role as key regulators of apoptosis and tumor suppression. PTEN is a well-established negative regulator of the PI3K-Akt signaling pathway, while BCL2L11 (BIM) functions as a critical pro-apoptotic protein (O’Connor et al. 1998; Vivanco & Sawyers 2002). In addition, SOCS1 and SOCS3 were identified as components of the network, acting as negative regulators of the JAK/STAT signaling pathway (Yoshimura et al. 2007). Given that ruxolitinib is a JAK inhibitor, the presence of these genes within the network suggests a potential mechanistic link between miRNA regulation and JAK/STAT pathway modulation.

Taken together, our findings suggest that TMZ and ruxolitinib combination treatment may reshape miRNA-associated regulatory networks in glioblastoma stem-like cells, particularly those linked to stemness, survival, inflammatory signaling, and therapy resistance. Rather than demonstrating direct mechanistic regulation, the present study supports a model in which JAK/STAT inhibition alters broader adaptive transcriptional programs that secondarily influence oncogenic miRNA expression patterns. In particular, hsa-miR-19a-3p, hsa-miR-221-3p, and hsa-miR-10a-5p emerge as potentially relevant components of resistance-associated regulatory networks. Future studies involving functional validation and mechanistic interrogation will be required to determine whether these miRNAs represent actionable mediators of combination therapy response in glioblastoma.

## Limitations

This study has several limitations that should be considered. First, the findings are based on in vitro experiments using glioblastoma cancer stem cells and therefore may not fully recapitulate the complexity of tumor behavior in vivo. In addition, the drug concentrations used in this study were determined under controlled in vitro conditions and may not directly correspond to clinically achievable exposure levels within the tumor microenvironment. Furthermore, pathway enrichment and network analyses were based on computational predictions and bioinformatic approaches, and thus require further experimental validation. Future studies incorporating in vivo models and functional assays are needed to confirm the biological relevance of the identified miRNAs, target genes, and associated signaling pathways.

## Conclusion

In conclusion, this study demonstrates that temozolomide treatment is associated with increased expression of oncogenic miRNAs, including hsa-miR-19a, hsa-miR-221, and hsa-miR-10a, while ruxolitinib treatment reduces their expression levels. The combination of ruxolitinib and temozolomide attenuated the TMZ-induced upregulation of these miRNAs. Bioinformatics analyses identified key target genes and signaling pathways involved in apoptosis and tumor progression, including PI3K-Akt, MAPK, and Wnt signaling pathways. Network analysis further highlighted central genes such as BCL2L11, PTEN, and SOCS family members.

These findings suggest that targeting miRNA-mediated regulatory networks may represent a promising strategy to overcome treatment resistance in glioblastoma.

## Data Availability

All source data for this work (or generated in this study) are available upon reasonable request.

## Abbreviations

BCSC: Brain cancer stem cell
BIM: Bcl-2 interacting mediator of cell death (BCL2L11)
DAVID: Database for annotation, visualization and integrated discovery
FC: Fold change
FDR: False discovery rate
GBM: Glioblastoma
JAK: Janus kinase
KEGG: Kyoto Encyclopedia of Genes and Genomes
MAPK: Mitogen-activated protein kinase
miRNA: MicroRNA
mTOR: Mechanistic target of rapamycin
PI3K: Phosphoinositide 3-kinase
PTEN: Phosphatase and tensin homolog
qRT-PCR: Quantitative real-time polymerase chain reaction
SOCS: Suppressor of cytokine signaling
STAT: Signal transducer and activator of transcription
TMZ: Temozolomide
